# “RAL” the LDLR to lysosomes for degradation

**DOI:** 10.1093/lifemeta/loag025

**Published:** 2026-08-21

**Authors:** Yangyang Guan, Yingzhen Huang, Zonghong Li, Yan Wang

**Affiliations:** Department of Cardiology of Zhongnan Hospital, State Key Laboratory of Metabolism and Regulation in Complex Organisms, Hubei Key Laboratory of Cell Homeostasis, TaiKang Center for Life and Medical Sciences, College of Life Sciences, Wuhan University, Wuhan, Hubei 430072, China; School of Biomedical Engineering, Guangzhou Medical University, Guangzhou, Guangdong 511436, China; Department of Cardiology of Zhongnan Hospital, State Key Laboratory of Metabolism and Regulation in Complex Organisms, Hubei Key Laboratory of Cell Homeostasis, TaiKang Center for Life and Medical Sciences, College of Life Sciences, Wuhan University, Wuhan, Hubei 430072, China; Guangzhou National Laboratory, Guangzhou, Guangdong 510005, China; Department of Cardiology of Zhongnan Hospital, State Key Laboratory of Metabolism and Regulation in Complex Organisms, Hubei Key Laboratory of Cell Homeostasis, TaiKang Center for Life and Medical Sciences, College of Life Sciences, Wuhan University, Wuhan, Hubei 430072, China

**Low-density lipoprotein receptor (LDLR)-dependent clearance of low-density lipoprotein (LDL) plays a pivotal role in maintaining cholesterol homeostasis. Widely used cholesterol-lowering therapeutics, such as statins and proprotein convertase subtilisin/kexin type 9 (PCSK9) inhibitors, exert their effects mainly by upregulating LDLR abundance and function. A recent study demonstrates that chronic high-cholesterol diet feeding activates Ral to drive lysosomal degradation of LDLR via a PCSK9-independent pathway, uncovering a novel therapeutic target for hypercholesterolemia and cardiovascular disease.** 

Cholesterol constitutes a fundamental building block of mammalian membranes, and cholesterol homeostasis is critical for proper cellular and organismal function. Cellular cholesterol pools are tightly monitored and delicately balanced by the rates of *de novo* synthesis, uptake, efflux, and esterification—a process either for storage in lipid droplets or for secretion in lipoproteins.

**Figure 1:**
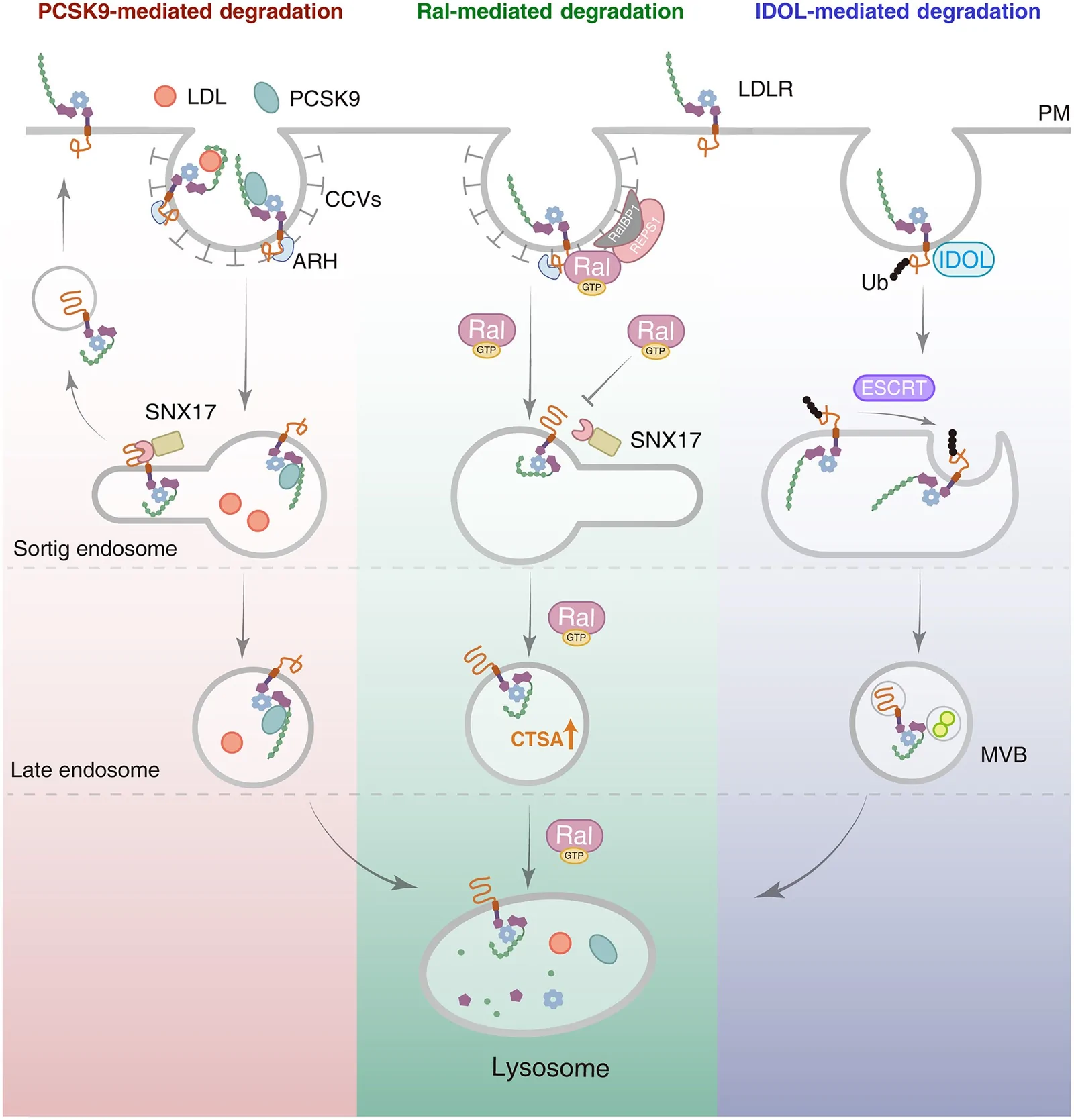
Lysosomal degradation of LDLR in hepatocytes. Cell-surface LDLR binds LDL and recruits the adaptor ARH via the cytoplasmic NPxY motif to enable internalization within clathrin-coated vesicles (CCVs). Within endosomes, acidic pH induces a conformational change in the LDLR extracellular domain, triggering dissociation from LDL particles. This conformational rearrangement propagates to the receptor’s cytoplasmic tail, strengthening its association with sorting nexin 17 (SNX17) and facilitating LDLR recycling through the same NPxY motif. LDL remains in the endosomal lumen and is degraded in lysosomes. PCSK9 binding inhibits acid-induced conformational remodeling and disrupts the LDLR–SNX17 interaction, preventing LDLR recycling to the cell surface and committing LDLR to lysosomal degradation (left). Ral activation drives lysosomal LDLR degradation via three distinct mechanisms (middle). At the plasma membrane, activated Ral promotes LDLR internalization through RalBP1 and REPS1; in sorting endosomes, activated Ral competes with SNX17 for LDLR binding to block receptor recycling; within lysosomes, Ral enhances CTSA maturation. IDOL acts as an E3 ubiquitin ligase to mediate LDLR ubiquitination. Ubiquitinated LDLR is targeted for lysosomal degradation via the endosomal sorting complex required for transport (ESCRT) pathway (right). Abbreviations: MVB: multivesicular body; PM: plasma membrane.

Since the 1930s, studies have demonstrated that cholesterol biosynthesis is subject to strict feedback regulation to maintain cholesterol homeostasis. Over the past 50 years, two major intracellular pathways governing cellular cholesterol homeostasis have been elegantly uncovered [1]. First, elevated cholesterol suppresses activation of sterol regulatory element-binding protein (SREBP), the master transcription factor controlling the entire cholesterol biosynthetic pathway, and also reduces the levels of low-density lipoprotein receptor (LDLR), the gatekeeper for cholesterol uptake. Second, excess cholesterol activates liver X receptor (LXR), another master transcription factor that drives cholesterol efflux primarily via ATP-binding cassette transporter A1 (ABCA1) and ATP-binding cassette sub-family G member 1 (ABCG1), as well as ATP-binding cassette sub-family G member 5/8 (ABCG5/8) in a cell type-specific manner. Meanwhile, LXR activation also facilitates LDLR degradation via an inducible degrader of LDLR (IDOL)-dependent ubiquitination and endosomal sorting complex required for transport (ESCRT) pathway [1] (Fig. 1). In a recent study, Feng *et al.* demonstrated that chronic cholesterol overload promotes LDLR degradation through a Ral-dependent pathway, providing novel insights into the regulation of cellular and systemic cholesterol homeostasis [2].

LDLR is a long-lived type 1 transmembrane protein that undergoes multiple rounds of internalization and recycling between the cell surface and endosomes via clathrin-mediated endocytosis [3] (Fig. 1). Low-density lipoprotein (LDL) binding to cell surface LDLR recruits the endocytic adapter autosomal recessive hypercholesterolemia protein (ARH) and disabled-2 (Dab2), as well as the associated adapter protein complex 2 (AP2) and clathrin, via the cytoplasmic NPxY motif and promotes LDLR internalization through the clathrin-coated vesicles (CCVs) [1]. In the acidic milieu of the endosome, the LDLR extracellular domain adopts a closed conformational change and dissociates from the LDL particles, with LDLR recycling back to the cell surface and LDL traveling to the lysosome for degradation [3]. A geometry-based endocytic sorting model provides a mechanistic explanation for this process: the LDLRs are preferentially enriched in the tubular structure of the sorting endosome because of its greater surface area to volume ratio, while the LDL particles are left within the vesicular lumen. After a period of time, the sorting endosome will stop accepting new early endosomes and mature into late endosomes and lysosomes. Thus, after multiple rounds of sorting, most of the LDLRs recycle back to the cell surface, whereas the LDLs are degraded in the lysosomes [4].

This geometry-based sorting mechanism indicates that receptors and cargo can be partitioned into distinct destinations via passive diffusion. Guan *et al.* recently uncovered that acidic pH, which mimics the luminal environment of sorting endosomes, drives a conformational rearrangement of the LDLR extracellular domain. This rearrangement further triggers a conformational shift in the cytoplasmic tail of the receptor, enhancing its binding to sorting nexin 17 (SNX17) and facilitating LDLR recycling through the NPxY motif [5] (Fig. 1). Proprotein convertase subtilisin/kexin type 9 (PCSK9), a circulating factor that promotes lysosomal degradation of LDLR, binds to the LDLR extracellular domain and blocks these acid-induced conformational changes as well as the LDLR–SNX17 interaction, ultimately targeting LDLR for lysosomal degradation (Fig. 1). Collectively, this work and previous findings support the following model governing LDLR trafficking regulation. At the plasma membrane, the LDLR extracellular domain adopts an open conformation competent for LDL binding, while its cytoplasmic tail engages ARH/Dab2 and clathrin via the NPxY motif to enable receptor internalization. After internalization into endosomes, acidic pH induces conformational changes in the LDLR ectodomain, promoting dissociation of LDL particles. This event concurrently remodels the cytoplasmic tail, releasing ARH and recruiting SNX17 to mediate endocytic sorting and LDLR recycling.

In their study, Feng *et al.* demonstrated that chronic high-cholesterol diet feeding enhances Ral activity via activated RAS signaling, which subsequently drives lysosomal degradation of LDLR in the liver. Mechanistically, activated Ral binds to LDLR at the same NPxY motif and impairs LDLR recycling by disrupting the interaction of the receptor with SNX17. In parallel, Ral activation facilitates LDLR internalization by recruiting its downstream effector Ral-binding protein 1 (RalBP1) and the endocytic protein RalBP1 associated EPS domain containing 1 (REPS1), and augments lysosomal function by promoting the maturation of cathepsin A (CTSA). Notably, rare coding variants in Kirsten rat sarcoma virus (*KRAS*), *REPS1*, and *CTSA* are associated with human blood lipid traits. Both Ral inhibition and CTSA blockade elevate LDLR abundance and reduce circulating cholesterol levels in mice fed a high-cholesterol diet. These findings reveal a promising new therapeutic strategy for hypercholesterolemia and cardiovascular disease.

This study also raises several intriguing questions. First, active Ral inhibits LDLR recycling by disrupting its interaction with SNX17, similar to PCSK9, yet via distinct mechanisms. Feng *et al.* demonstrated that Ral drives LDLR degradation independently of PCSK9, and PCSK9 promotes LDLR degradation independently of Ral activation as well. How PCSK9 and Ral activation coordinately regulate SNX17 binding and LDLR recycling remains to be clarified.

Second, Ral activation on the plasma membrane facilitates LDLR internalization. Whether this process depends on direct binding between Ral and LDLR remains unknown. ARH and Dab2 are essential adaptors that mediate LDLR internalization through binding to the same NPxY motif. Notably, chronic high-cholesterol diet feeding suppresses hepatic ARH expression in mice, implying potential functional crosstalk between Ral-dependent and ARH-mediated LDLR internalization, which merits further investigation. Unlike Ral activation, however, PCSK9 does not promote LDLR internalization based on direct measurement of the LDLR internalization rate from the cell surface [5].

Third, this work reveals that Ral activation enhances CTSA maturation and lysosomal function to specifically promote LDLR breakdown, offering new insights into the control of LDLR stability. Guan *et al.* did not directly assess lysosomal activity following PCSK9 treatment. Nevertheless, the finding that PCSK9 fails to accelerate degradation of recycling-deficient LDLR mutants renders this scenario less plausible. How Ral activation governs CTSA maturation also remains unresolved. Ral is ubiquitously expressed. Whether Ral activation controls LDLR degradation in tissues and cell types beyond the liver and hepatocytes remains an open question.

Ral is well characterized as a regulator of vesicle trafficking and exocytosis. The present study broadens Ral function to encompass receptor internalization, endocytic sorting, and lysosomal degradation, three core steps of the receptor-mediated lysosomal degradation pathway originally established by Brown and Goldstein in the 1970s. Intriguingly, Ral activation selectively targets LDLR for degradation and does not affect other receptors bearing the conserved NPxY motif, such as epidermal growth factor receptor (EGFR). Numerous receptors contain NPxY sequences, including LDLR superfamily members, integrin β subunits, amyloid precursor protein, and others. Whether Ral activation modulates the stability of these receptors awaits future exploration. Fully deciphering the molecular basis underlying LDLR-specific regulation by Ral will be critical for advancing translational research.

In summary, Feng *et al.* describe a previously unrecognized cascade governing LDLR stability. Together with supporting human genetic evidence, these findings uncover a promising therapeutic strategy for hypercholesterolemia and cardiovascular disease.
